# Population genetic portrait of Pakistani Lahore-Christians based on 32 STR loci

**DOI:** 10.1038/s41598-020-76016-2

**Published:** 2020-11-03

**Authors:** Aqsa Rubab, Muhammad Shafique, Faqeeha Javed, Samia Saleem, Fatima Tuz Zahra, Dennis McNevin, Ahmad Ali Shahid

**Affiliations:** 1 Forensic DNA Typing Laboratory, Centre of Excellence in Molecular Biology, University of the Punjab, Lahore, 53700 Pakistan; 2 School of Biomedical Sciences, The University of Hong Kong, Pok Fu Lam, Hong Kong; 3 Centre for Forensic Science, University of Technology Sydney, Ultimo, Australia

**Keywords:** Biological techniques, Computational biology and bioinformatics, Genetics, Molecular biology

## Abstract

Phylogenetic relationship and the population structure of 500 individuals from the Christian community of Lahore, Pakistan, were examined based on 15 autosomal short tandem repeats (STRs) using the AmpFℓSTR Identifiler Plus PCR Amplification Kit and our previously published Y-filer kit data (17 Y-STRs) of same samples. A total of 147 alleles were observed in 15 loci and allele 11 at the TPOX locus was the most frequent with frequency value (0.464). The data revealed that the Christian population has unique genetic characteristics with respect to a few unusual alleles and their frequencies relative to the other Pakistani population. Significant deviations from Hardy–Weinberg equilibrium were found at two loci (D13S317, D18S51) after Boneferroni’s correction (*p* ≤ 0.003). The combined power of discrimination, combined power of exclusion and cumulative probability of matching were 0.999999999999999978430815060354, 0.999995039393942 and 2.15692 × 10^−17^, respectively. On the bases of genetic distances, PCA, phylogenetic and structure analysis Lahore-Christians appeared genetically more associated to south Asian particularly Indian populations like Tamil, Karnataka, Kerala and Andhra Pradesh than rest of global populations.

## Introduction

Pakistan is a multiethnic country, harboring 217 million people, of whom the majority is Muslim according to the Pakistan Burea of Statistics^1^. Minority religious affiliates residing in Pakistan include Hindus, Christians, Ahmedis, Baha'is, Sikhs, Parsis, and Buddhists, amongst others. The Christian population comprises of 2.5 million (1.6%), making it the second largest religious minority of Pakistan^2^. Lahore, the capital of the Pakistani province of Punjab, is the second-most populous city in Pakistan (11.13 million) with a Muslim majority (97%) and a Christian minority (2%). Christianity was initially imported by Reverend Thomas Valpy who was appointed as the first Bishop of Lahore in 1877^3^. Christians are considered to be descendants of a caste population of India^4^ and while they are thought to be a relatively closed population because of religious constraints, yet amiable relations are sustained with the majority population.

Short tandem repeats (STRs), also known as microsatellites, are repetitive sequences of DNA with a repeat motif of four to six base pairs and are almost universally employed as forensic identity markers because they are highly polymorphic and heterozygous, have short sequence lengths and are distributed throughout the human genome^5,6^ Although their mutation rates are significantly higher than those for single nucleotide polymorphisms (SNPs)^7^, they are none the less useful as genetic markers for population genetic studies, especially more recent genetic history^8^.

There have been many earlier studies of 15 autosomal STRs in various Pakistani populations except Christians. We emphasize that this population must be targeted as a whole, to understand the genetic context of Christians and its connection to the greater Eurasian continent. Hence, Lahore-Christian samples were evaluated based on fifteen autosomal STRs of Identifiler Plus Kit (Applied Biosystems) and already published data set of same male samples (YA004381)^9^ on 17 YSTRs (DYS438, DYS393, DYS385a⁄b, DYS389I⁄II, DYS458, DYS437, DYS391, DYS392, DYS635 (Y-GATA-C4), Y-GATA-H4, DYS19, DYS390, DYS439, DYS456, DYS448). To affirm phylogenetic affiliations of this population, data sets were compared with referenced populations as given in Table 1.

**Table 1:** Datasets for various analyses in this study.

Dataset	Analysis	Population	Geographic regions	Data	References
Dataset I	PCA, phylogenetic tree, population differentiation test	Christians	Pakistan	Autosomal STRs	This study
		Punjabi			^14^
		Sindhi			^15^
		Kashmiri			^16^
		Balochi			^17^
		Yousafzai			^18^
		Tamil	South India		^19^
		Kerala	South India		^20^
		Karnataka	South India		^21^
		Balmiki	North India		^22^
		Madhya Pradesh	Central India		^23^
		Nepal	South Asia		^24^
		Bangladeshi			^25^
		Mongol	East Asia		^26^
		Caucasian	Europe		^27^
		Uganda	Africa		^28^
		African American			^27^
	Structure	Christians	Pakistan		This study
		Punjabi			^14^
		Sindhi			^15^
		Tamil	South India		^19^
		Mongol	East Asia		^26^
		Caucasian	Europe		^27^
		Romania			^29^
		AfricanAmerican	Africa		^27^
Dataset II	Neighbour joining tree, MDS	Christians	Pakistan	YSTRs	YHRD
		Punjabi			
		Sindhi			
		Kashmiri			
		Balochi			
		Yousafzai			
		Tamil	South India		
		Balmiki	North India		
		Madhya Pradesh	Central India		
		Andhra Pradesh	South India		
		Karnataka	South India		
		Nepal	South Asia		
		Bangladeshi			
		Mongol	East Asia		
		Caucasian	Europe		
		Uganda	Africa		
		African American			
	Haplogroup	Christians	Pakistan		^9^

## Materials and methods

### Sample collection

About 3 mL blood was collected in EDTA vacutainer tubes from 500 unrelated Christian individuals residing Lahore, capital city of the Punjab province in Pakistan. Whatman blood stain cards were prepared for each sample with a unique sample ID that was henceforth used for processing.

### DNA extraction and quantitation

Genomic DNA was isolated by an organic-extraction procedure described by Signer et al. (1988)^10^ and quantified on ABI7500 Real-Time PCR instrument (Applied Biosystems) using the Quantifiler Human DNA Quantification Kit (Applied Biosystems) following the recommended protocol^11^.

### Amplification

DNA samples were diluted to the concentration of 1 ng/μL for PCR according to the recommended protocol for the AmpFℓSTR Identifiler Plus PCR Amplification Kit (Applied Biosystems)^12^. The DNA template (1 ng) was added to 2.4μL of Master Mix and 1.2μL Primer Set in a total reaction volume of 6 µL. PCR was performed in a GeneAmp9700 PCR System (Applied Biosystems). Thermal cycler conditions included an initial incubation for 11 min at 95 °C; 28 cycles of denaturation for 20 s at 94 °C, annealing/extension for 3 min at 59 °C and final extension for 10 min at 60 °C; and a final hold at 4 °C.

### Genotyping

To perform genotyping on an ABI3730xl Genetic Analyzer (Applied Biosystems), 1µL of amplified product was added to 0.35µL GeneScan 500 LIZ size standard (Applied Biosystems) and 13µL highly deionized (Hi-Di) formamide. Data was analyzed using GeneMapper ID v3.2 to designate alleles in accordance with the Kit allelic ladder.

### Quality control

The efficiency of the PCR amplification was monitored using Identifiler Plus Control DNA 9947A as a positive control and all reagents except DNA template as negative control. The STR analysis was conducted following the nomenclature recommendations of the DNA Commission of the International Society for Forensic Genetics (ISFG)^13^. The dataset was evaluated by the STRidER database^13^ with QC report reference number STR000284.

### Population datasets used for comparison

The STR data of Lahore-Christians was compared with the available data of indigenous and global populations (supplementary Table 1) derived from published sources as summarized in Table 1.

### Data analysis

Statistical parameters of forensic interest including power of discrimination (PD), matching probability (MP), observed (*H*_O_) and expected (*H*_E_) heterozygosities, polymorphism information content (PIC), typical paternity index (TPI), power of exclusion (PE) and allele frequencies were determined using modified Powerstats1.2 software^30^. A Hardy Weinberg equilibrium (HWE) test was performed using PowerMarker3.25^31^. The exact test for population differentiation was carried out by Arlequin3.5.2.2 software^32^. Phylogenetic and Principal Component analysis were executed using POPTREE^33^, MEGA-X^34^, Structure2.3.4^35^ and PAST3.26^36^. Y-DNA haplogroups were also predicted by Whit Athey’s Haplogroup Predictor (https://www.hprg.com/hapest5/index.html)^37^ for the purpose of Y-lineage identification.

### Ethical approval

All participants were introduced to this study and blood samples were collected with their Informed consent. The study was carried out in accordance with the relevant guidelines and regulations approved by the Ethical Committee of the Centre of Excellence in Molecular Biology, University of Punjab Lahore Pakistan (No. CEMB/AO/2289).

## Results and discussion

### Allelic frequencies and forensic parameters

A total of 147 alleles were observed over all loci and allele 11 at the TPOX locus was found to have the highest frequency of 0.46. Allelic frequencies at each locus are shown in Supplementary Table 2 while the parentage and forensic statistical parameters are in Supplementary Figure 1. Supplementary Table 3 shows five uncommon alleles (UCA) observed, together with most and least common alleles at each locus. Few alleles like 12.2, 14.2, 15.2, 16.2 at D19S433 and 9.1 at D7S820 were also reported to NIST STR Database. Polymorphism information content (PIC) was in the range of 0.623 (CSF1PO) to 0.841 (FGA) and the most discriminating marker was FGA with a PD value of 0.961. The observed heterozygosity varied from 0.656 (TPOX) to 0.868 (D8S1179) and the power of exclusion (PE) ranged from 0.364 (TPOX) to 0.731 (D8S1179). The power of discrimination (CPD), combined power of exclusion (CPE) and combined probability of matching (CPM) were 0.999999999999999978430815060354, 0.999995039393942 and 2.15692 × 10^−17^, respectively. Significant deviations from Hardy–Weinberg equilibrium (*p* < 0.05) were observed at two loci (D13S317, D18S51) after Boneferroni’s correction (*p* ≤ 0.003).

### Interpopulation comparison

The allele frequencies at the 15 autosomal STRs in the Lahore-Christian population were compared with those from 16 other populations using population differentiation test as shown in Supplementary Table 4. Significant differences were observed after Boneferroni’s correction (*p* ≤ 0.0002) at 15/15 loci with Mongol, 14/15 African American^26,27^, Caucasian (12/15)^27^, Uganda Yousafzai and Kashmiri (11/15)^16,18,28^, Balochi, Punjabi, central India (7/15)^14,23,38^. While differences at small numbers of loci for Nepalese, Sindhi (3/15), Karnataka and Bangladeshi (2/15)^21,24,25,39^ were observed. However, there were no significant differences at any loci for the Tamil, Balmiki, and Kerala populations^19,20,22^.

### Phylogenetic analysis

**Figure 1:**
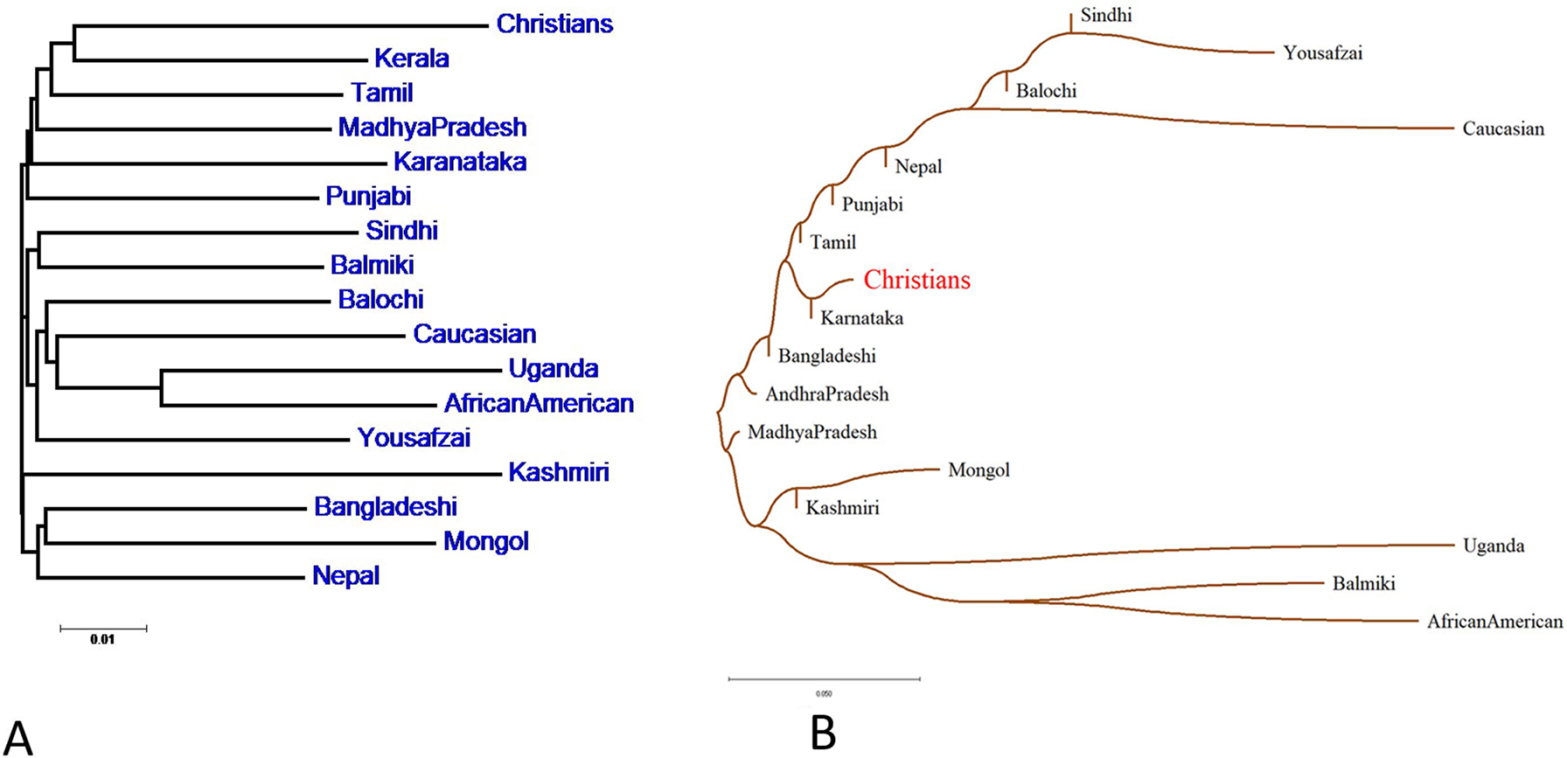
(**A**) Phylogenetic tree constructed using POPTREE based on Fst corrected values of 15 autosomal STRs in the Lahore-Christians and 16 other populations. (**B**) A neighbour joining tree generated with MEGA-X software based on R_ST_p-values of17 YSTR in Lahore-Christians and 16 reference populations.

The neighbour-joining phylogenetic tree (Fig. 1A) illustrates genetic relationships between the Lahore-Christian population and 16 reference populations based on Fst corrected values of 15 autosomal STRs. Phylogenetic tree showed that Lahore-Christians appeared most closely associated to South Indians like Kerala and Tamil followed by Madhya Pradesh (Central Indian), Karnataka (Iyengar Brahmin) and Pakistani Punjabi. Other Pakistani Populations were distantly associated like Sindhi grouped with North Indian Balmiki; Balochi and Yousafzai Pathan shared genetic association to Caucasian, Uganda and African American. Similarly, a neighbour joining tree was constructed using our published 17-YSTRs data^9^ of studied population and 16 reference populations based upon R_ST_ p-values. Pairwise R_ST_ p-values were calculated through AMOVA using online YHRD tool. As illustrated in Fig. (1B) paternal lineage of Lahore-Christians shared branch with South Indian-Karnataka adjoining roots with Tamils, Andhra Pradesh and Bangladeshi followed by Punjabi. While Sindhi, Yousafzai and Balochi shared genetic association with Caucasians at the top. However, Madhya Pradesh (Central Indian) appeared distantly. Phylogenetic analysis shows Lahore-Christians population has a close genetic distance with the south Indian populations.

South India accounts for 21.47% of the community. It experienced a range of cross-cultural challenges between missionary Christianity and local converts^40^. Historically, the Tamil, Kerala and Karnataka populations belonged to the southern part of India and their culture is deeply rooted in Christians and Muslims^41^. Kerala is home to 22.07% of the total Christians in the country, followed by Tamil Nadu with 15.88%^42^. According to a 2011 census, Christians represent about 6% of the Tamil Nadu state population^43^ which also proclaim our phylogenetic analysis and migration history of Lahore-Christians. Moreover, it suggests that while South Indians and Pakistani Christians are geographically isolated, they have similar genetic origins.

### Structure analysis

**Figure 2:**
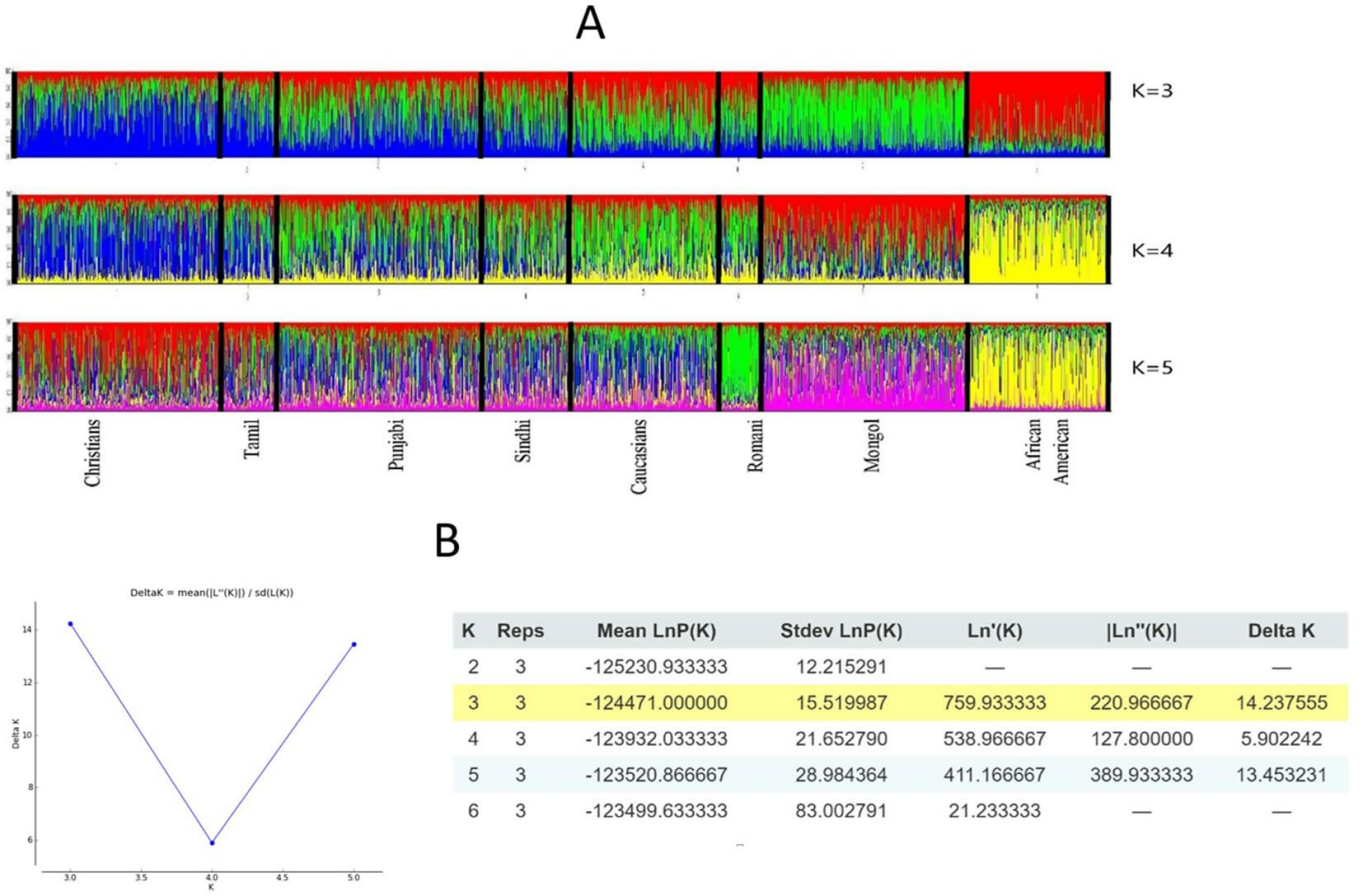
(**A**) Bar plot representing structure analysis of Lahore-Christians in comparison to 7 other populations based on 15 autosomal STRs. (**B**) Illustrates maximum of delta *K* and evanno table values.

Although 15 autosomal STR markers have limited differentiation power to detect population structure but are efficient to some extent in differentiating Lahore-Christians from 9 other reference populations. Structure analysis was conducted employing Structure2.3.4 software using the admixture model with correlated allele frequencies without prior population information (USEPOPINFO = 0). Number of inferred clusters varied from 1 to 6 with three repetitions using 50,000 burnin and 100,000 MCMC simulation for each K. Results are intuitively depicted by bar plot as illustrated in Fig. 2A. All populations were partitioned into K colored segments depending on the value of K.

Whereas, K = 3 was the most suitable configuration based upon output posterior probability results inferred using the Structure Harvester^44^ as depicted in Fig. 2B. At K = 3 African American and Mongol were almost entirely filled with red and green component respectively. Lahore-Christians and Tamil shared blue color as major component structure in similar pattern that gradually diminished in next populations. Punjabi and Sindhi presented the mixture of green and blue components whereas Europeans (Caucasian, Romani) shared a mixture of red and green component to similar extent. While we may have expected Christians to exhibit some differentiation from the other Pakistani populations, it is not surprising that Lahore-Christians and South Asian Tamil are not differentiated by the STRs in the Identifiler panel using Structure.

### Principal components analysis

**Figure 3:**
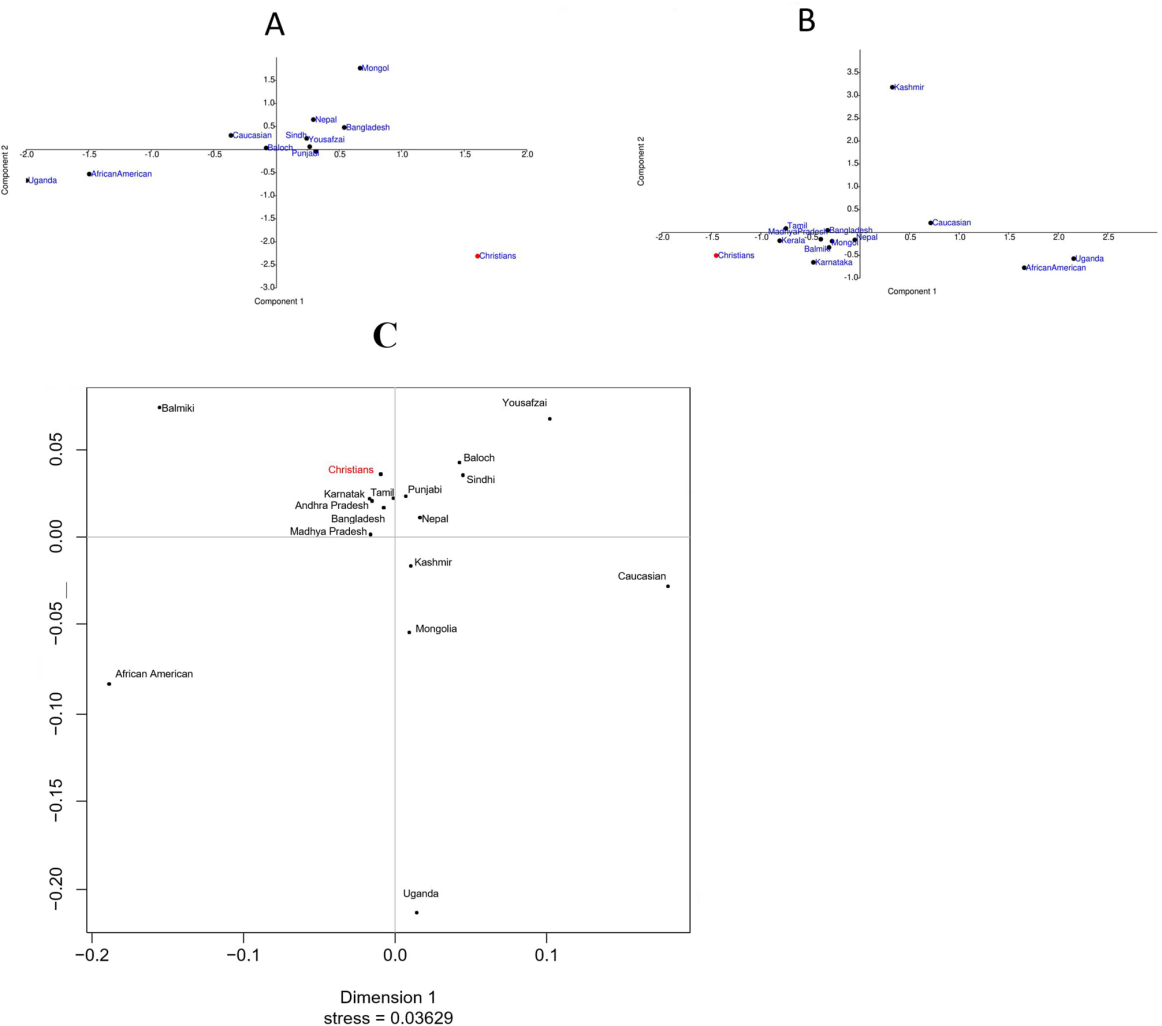
(**A**) Principal component analysis (PCA) plot constructed from allele frequencies of 15 autosomal STR loci in the Lahore-Christian population, 4 indigenous populations (Punjabi, Sindhi, Yousafzai, Baloch) and 6 other populations. (**B**) PCA plot based upon allele frequencies of studied population, 5 Indian and 7 other reference populations. (**C**)The Multidimensional scaling (MDS) plot showed genetic relationships between Lahore-Christians, Pakistani, Indians and other populations.

A PCA plot was constructed from autosomal STR allele frequencies (Supplementary Table 1) among Lahore-Christians, 4 indigenous reference populations (Fig. 3A) and global populations (Fig. 3B). In Fig. 3A Lahore-Christians signified as divergent population in lower right quadrant while rest of Pakistani populations clustered in upper right quadrant. Other global populations were scattered in the plot. In Fig. 3B Lahore-Christians were compared to Indian and 7 other world populations. It shows that studied population is relatively closer to South Indian populations (Karnataka, Kerala and Tamil) as compared to others. In Fig. 3A,B components 1 and 2 explain 55% and 46% of the variance respectively indicating genetic distances between populations.

Multidimensional scaling plot was generated based on haplotype data of YSTRs, to figure out Lahore-Christians paternal lineage (Fig. 3C). In this plot the Lahore-Christians remained tightly clustered with South Indian (Tamil, Karnatka, Andhra Pradesh), Central Indians (Madhya Pradesh) and Bangladeshi population. Balmiki was found distantly associated in the same quadrant whereas other populations including Pakistani, European American, Mongol, African American and Uganda scattered in different quadrants.

### Y-DNA haplogroups

Haplotypes of Lahore Christians (n = 250) based on 17 –YSTRs using Whit–Athey’s algorithm were assigned to 7 haplogroups (L, Q, R, E1b1b, G2a, J2a1b, J2a1 x J2a1-bh). Other haplogroups like I1, G2c, I2a1, J2a1h I2b1, N and T were not observed in our samples. Whereas, L(40%), RIa(38%), E1b1b(25%), Q(23%) were found the most common haplogroups and accounted for most of its Y-lineage from south Asians. Our results also corroborated with the past reportings of the most frequent haplotypes from South Asia^45^.

The outcomes of phylogenetic analysis presented that Lahore Christians are most closely related to Indians particularly Tamil and might share common ancestors. Moreover, there are clear genetic variations between Christians and rest of the populations. It also supports historical records that, following the geographical migration from India to Pakistan, this population got eventually recognized as Christians^46^.

Lahore-Christians are primarily nomadic, poses conservative lifestyle, religious practices, extremely endogamous culture and traditional occupation as compared to other Pakistani population. Tracing their trail of migration and relatedness with world populations would provide a glimpse of primordial trajectory. Genetic affinity of Lahore-Christians to South Asian Indian populations and their common nomadic practices indicates historical genetic relatedness. Migratory events lead to subsequent separation of both populations. Relatively higher genetic distance to other Pakistani population were observed in our current study. Previous reports have also suggested genetic similarities of Tamils representing their common origin but minimal signature of gene exchange with other nomadic groups^47^.

However there were certain inconsistencies seen in side by side comparison at fewer population groups based on autosomal and YSTRs due to limited availability of their respective samples data. However, all these analyses clearly indicate that Lahore-Christian population has close genetic affiliation to South Indian population. Moreover, significant differences were observed between Lahore-Christians and other Pakistani populations except Punjabi that seems bit closer. This might also indicate that Lahore-Christians and Pakistani Punjabi diverged gradually from native South Indians following its geographical migration, which also corresponded with historical records^48^.

## Conclusion

We have provided evidence that the Christian population in Lahore, Pakistan, forms a sub-population among Asian groups and has some unique genetic characteristics^14,39,49^. Results of inter-population differentiations, PCA, phylogenetic and structure analysis revealed that Lahore Christians have relatively close genetic relationships with south Asians particularly Indians. Being closely related to South Indians therefore it showed close resemblance to Tamil, Kerala, Andhra Pradesh and Karnataka populations. In this population, the 15 autosomal STRs and 17 YSTRs provide ample information for lineage characterization. This data would be useful for studies of genealogy, historical migration of Pakistani populations and database development. Genetic data obtained from autosomal and YSTR are in accord with human migration history of Indo-Pak populations. However, there is need of a detailed mitochondrial study to assign them mitochondrial haplogroups for maternal lineage identification.

## Supplementary information

Supplementary Information 1.

Supplementary Information 2.

Supplementary Information 3.

Supplementary Information 4.

Supplementary Information 5.

## References

[1] Statistics, F. B. O. Government of Pakistan. *Change* (2007).

[2] Victor DG, House JC, Joy S (2005). A Madisonian approach to climate policy. Science.

[3] Hassan F (2006). Pakistan's federal structure and the constitution of 1973. Muslim World.

[4] Pio, E. & Syed, J. in *Faith-Based Violence and Deobandi Militancy in Pakistan* 187–207 (Springer, 2016).

[5] Ellegren H (2004). Microsatellites: simple sequences with complex evolution. Nat. Rev. Genet..

[6] Willems T (2014). The landscape of human STR variation. Genome Res..

[7] Sun JX (2012). A direct characterization of human mutation based on microsatellites. Nat. Genet..

[8] Phillips C (2018). Global patterns of STR sequence variation: sequencing the CEPH human genome diversity panel for 58 forensic STRs using the illumina ForenSeq DNA signature prep kit. Electrophoresis.

[9] Saleem S (2019). Phylogenetic analysis and haplotype diversity in Christian residents of Lahore, Pakistan, using 17 Y-chromosomal STR loci. Int. J. Leg. Med..

[10] Signer E, Kuenzle CC, Thomann PE, Hübscher U (1988). DNA fingerprinting: improved DNA extraction from small blood samples. Nucleic Acids Res..

[11] Barbisin M (2009). Developmental validation of the Quantifiler Duo DNA Quantification Kit for simultaneous quantification of total human and human male DNA and detection of PCR inhibitors in biological samples. J. Forensic Sci..

[12] Applied Biosystems AmpFℓSTR Identifiler Plus PCR Amplification Kit—User Guide (2018). ThermoFisher Scientific, UK.

[13] Bodner M (2016). Recommendations of the DNA Commission of the International Society for Forensic Genetics (ISFG) on quality control of autosomal Short Tandem Repeat allele frequency databasing (STRidER). Forensic Sci. Int. Genet..

[14] Shafique M (2015). Genetic diversity of 15 autosomal STR loci in the population of Southern Punjab Pakistan. Forensic Sci. Int. Genet..

[15] Perveen R (2018). Forensic and Phylogenetic Characterization of Pakistani Population Using Uniparental and Biparental Genetic Markers.

[16] Mohapatra B, KamalChauhan U, Thakur BY (2016). Anupuma Raina. Genetic analysis and evolutionary relationship of Jammu and Kashmir Muslim population with short tandem repeat loci. Int. J. Curr. Res..

[17] Khan AA (2019). Genetic polymorphism of 15 autosomal short tandem repeats in Baloch population of Pakistan. Int. J. Leg. Med..

[18] Batool Z (2018). Genetic analysis of 15 autosomal STRs in Yousafzai population of Pakistan. Int. J. Leg. Med..

[19] Balamurugan K (2010). Genetic variation of 15 autosomal microsatellite loci in a Tamil population from Tamil Nadu, Southern India. Leg. Med..

[20] Sreekumar R (2020). Allelic frequency database of 15 polymorphic autosomal STRs in the Malayalam-speaking population of Kerala, India. Int. J. Leg. Med..

[21] Rajkumar R, Kashyap V (2002). Distribution of alleles of 15 STR loci of the Powerplex 16 multiplex system in four predominant population groups of South India. Forensic Sci. Int..

[22] Ghosh T (2011). Genetic diversity of autosomal STRs in eleven populations of India. Forensic Sci. Int. Genet..

[23] Shrivastava P, Jain T, Trivedi VB (2015). Genetic polymorphism study at 15 autosomal locus in central Indian population. SpringerPlus.

[24] Ota M (2007). Allele frequencies for 15 STR loci in Tibetan populations from Nepal. Forensic Sci. Int..

[25] Hossain T (2016). Genetic polymorphism studies on 22 autosomal STR loci of the PowerPlex Fusion System in Bangladeshi population. Leg. Med..

[26] Zhan X (2018). Forensic characterization of 15 autosomal STRs in four populations from Xinjiang, China, and genetic relationships with neighboring populations. Sci. Rep..

[27] Hill CR, Duewer DL, Kline MC, Coble MD, Butler JM (2013). US population data for 29 autosomal STR loci. Forensic Sci. Int. Genet..

[28] Gomes V (2009). Population data defined by 15 autosomal STR loci in Karamoja population (Uganda) using AmpF/STR Identifiler kit. Forensic Sci. Int. Genet..

[29] Anghel A (2014). Genetic polymorphism data on 15 autosomal STR markers in a Western Romanian population sample. Leg. Med..

[30] Tereba A (1999). Tools for analysis of population statistics. Profiles DNA.

[31] Liu K, Muse SV (2005). PowerMarker: an integrated analysis environment for genetic marker analysis. Bioinformatics.

[32] Excoffier L, Lischer HE (2010). Arlequin suite ver 3.5: a new series of programs to perform population genetics analyses under Linux and Windows. Mol. Ecol. Resour..

[33] Takezaki N, Nei M, Tamura K (2009). POPTREE2: Software for constructing population trees from allele frequency data and computing other population statistics with Windows interface. Mol. Biol. Evol..

[34] Kumar S, Stecher G, Li M, Knyaz C, Tamura K (2018). MEGA X: molecular evolutionary genetics analysis across computing platforms. Mol. Biol. Evol..

[35] Pritchard, J. K., Wen, W. & Falush, D. Documentation for STRUCTURE software: Version 2. (2003).

[36] Hammer O, Harper DA, Ryan PD (2001). PAST: paleontological statistics software package for education and data analysis. Palaeontol. Electron..

[37] Athey TW (2006). Haplogroup prediction from Y-STR values using a Bayesian-allele-frequency approach. J. Genet. Geneal.

[38] Khan, A. A. & Perveen, R. Nadeem Sheikh, Babar Hilal Ahmad Abbasi, Zunaira Batool, Muhammad Shahzad & Sana Kaleem.

[39] Perveen R, Shahid AA, Shafique M, Shahzad M, Husnain T (2017). Genetic variations of 15 autosomal and 17 Y-STR markers in Sindhi population of Pakistan. Int. J. Leg. Med..

[40] Doss MC (2018). Indian Christians and the making of composite culture in South India. South Asia Res..

[41] Bayly S (2004). Saints, Goddesses and Kings: Muslims and Christians in South Indian Society, 1700–1900.

[42] Reich D, Thangaraj K, Patterson N, Price AL, Singh L (2009). Reconstructing Indian population history. Nature.

[43] Collins PM (2016). Christian Inculturation in India.

[44] Evanno G, Regnaut S, Goudet J (2005). Detecting the number of clusters of individuals using the software STRUCTURE: a simulation study. Mol. Ecol..

[45] Mahal DG, Matsoukas IG (2018). The geographic origins of ethnic groups in the Indian subcontinent: exploring ancient footprints with Y-DNA haplogroups. Front. Genet..

[46] Pervaiz H, Mahmood T (2018). Mass conversion to Christianity: A case study of Chuhra Community in Sialkot Distric (1880–1930). Pak. Vis..

[47] Watkins W (2008). Genetic variation in South Indian castes: evidence from Y-chromosome, mitochondrial, and autosomal polymorphisms. BMC Genet..

[48] Visaria PM (1969). Migration between India and Pakistan, 1951–61. Demography.

[49] Ali N, Coulson-Thomas YM, Dixon RA, Williams DR (2014). Genetic variation comparison of 15 autosomal STR loci in an immigrant population living in the UK (British Pakistanis) with an ancestral origin population from Pakistan. Forensic Sci. Int. Genet..

