# Supplementary material for: Population genetic portrait of Pakistani Lahore-Christians based on 32 STR loci
Source: Sci Rep. 2020 Nov 3;10:18960. doi: 10.1038/s41598-020-76016-2 (PMC7609739; doi:10.1038/s41598-020-76016-2)
Supplement: Supplementary file 1 — Supplementary Information 1. [file 41598_2020_76016_MOESM1_ESM.docx]

# Population genetic portrait of Pakistani Lahore-Christians based on 32 STR loci

Aqsa Rubab^1^, Muhammad Shafique^1*^, Faqeeha Javed^1^, Samia Saleem^1^, Fatima Tuz Zahra^2^, Dennis McNevin^3^, Ahmad Ali Shahid^1^

^1^ Forensic DNA Typing Laboratory, Centre of Excellence in Molecular Biology, University of the Punjab Lahore Pakistan-53700.

^2^ School of Biomedical Sciences, The University of Hong Kong, Hong Kong.

^3^ Centre for Forensic Science, University of Technology Sydney, Australia.


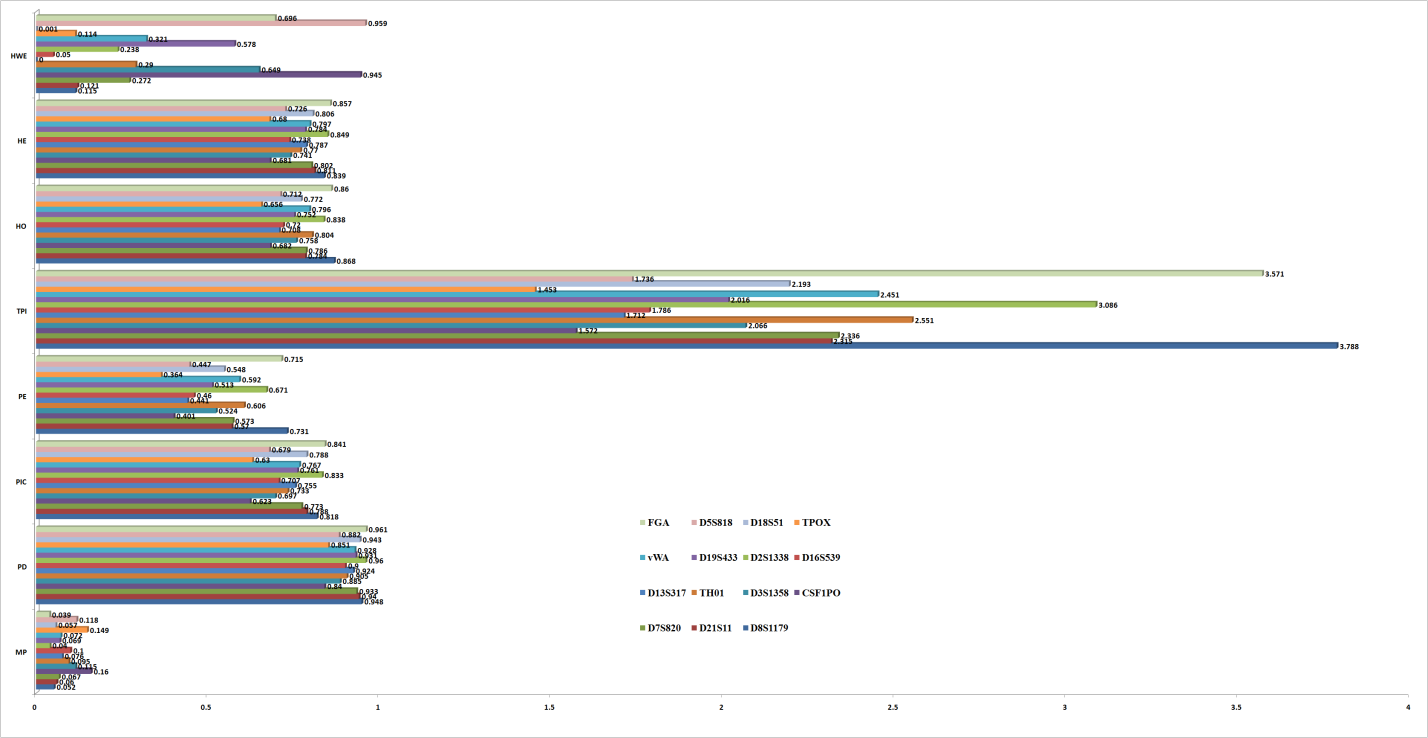


Supplementary Figure 1: Forensic efficiency parameters summarized for 15 autosomal STRs in the Christian population of Lahore, Pakistan. MP: matching probability; PD: power of discrimination; PIC: polymorphism information content; PE: power of exclusion; TPI: typical paternity index; H_O_: observed heterozygosity; H_E_: expected heterozygosity; HWE: Hardy Weinberg equilibrium.
